# Purple-grained barley (*Hordeum vulgare* L.): marker-assisted development of NILs for investigating peculiarities of the anthocyanin biosynthesis regulatory network

**DOI:** 10.1186/s12870-019-1638-9

**Published:** 2019-02-15

**Authors:** Elena I. Gordeeva, Anastasiya Yu. Glagoleva, Tatjana V. Kukoeva, Elena K. Khlestkina, Olesya Yu. Shoeva

**Affiliations:** 1 Institute of Cytology and Genetics, Siberian Branch of the Russian Academy of Sciences, Lavrentjeva ave. 10, Novosibirsk, 630090 Russia; 2 Novosibirsk State University, Pirogova str., 1, Novosibirsk, 630090 Russia; 3 N.I. Vavilov All-Russian Research Institute of Plant Genetic Resources (VIR), St. Petersburg, 190000 Russia

**Keywords:** *Hordeum vulgare*, Microsatellites, Near isogenic lines, Transcription factors, Structural genes, *Ant1*, *Ant2*, Positive feedback

## Abstract

**Background:**

Anthocyanins are plants secondary metabolites important for plant adaptation to severe environments and potentially beneficial to human health. Purple colour of barley grain is caused by the pigments synthesized in pericarp. One or two genes determine the trait. One of them is *Ant2* mapped on chromosome 2HL and is known to encode transcription factor (TF) with a bHLH domain. In plants, bHLH regulates anthocyanin biosynthesis together with TF harboring an R2R3-MYB domain. In wheat, the R2R3-MYBs responsible for purple colour of grain pericarp are encoded by the homoallelic series of the *Pp-1* genes that were mapped on the short arms of chromosomes 7. In barley, in orthologous positions to wheat’s *Pp-1*, the *Ant1* gene determining red colour of leaf sheath has been mapped. In the current study, we tested whether *Ant1* has pleiotropic effect not only on leaf sheath colour but also on pericarp pigmentation.

**Results:**

А set of near isogenic lines (NILs) carrying different combinations of alleles at the *Ant1* and *Ant2* loci was created using markers-assisted backcrossing approach. The dominant alleles of both the *Ant1* and *Ant2* genes are required for anthocyanin accumulation in pericarp. A qRT-PCR analysis of the *Ant* genes in lemma and pericarp of the NILs revealed that some reciprocal interaction occurs between the genes. Expression of each of the two genes was up-regulated in purple-grained line with dominant alleles at the both loci. The lines carrying dominant allele either in the *Ant1* or in the *Ant2* locus were characterized by the decreased level of expression of the dominant gene and scant activity of the recessive one. The *Ant1* and *Ant2* expression was barely detected in uncolored line with recessive alleles at both loci. The anthocyanin biosynthesis structural genes were differently regulated: *Chs*, *Chi*, *F3h*, *Dfr* were transcribed in all lines independently on allelic state of the *Ant1* and *Ant2* genes, whereas *F3’h* and *Ans* were activated in presence on dominant alleles of the both regulatory genes.

**Conclusions:**

The R2R3-MYB-encoding counterpart (*Ant1*) of the regulatory *Ant2* gene was determined for the first time. The dominant alleles of both of them are required for activation of anthocyanin synthesis in barley lemma and pericarp. The R2R3-MYB + bHLH complex activates the synthesis via affecting expression of the *F3’h* and *Ans* structural genes. In addition, positive regulatory loop between *Ant1* and *Ant2* was detected. Earlier the interaction between the anthocyanin biosynthesis regulatory genes has been revealed in dicot plant species only. Our data demonstrated that the regulatory mechanism is considered to be more common for plant kingdom than it has been reported so far.

**Electronic supplementary material:**

The online version of this article (10.1186/s12870-019-1638-9) contains supplementary material, which is available to authorized users.

## Background

Anthocyanins are flavonoid pigments that accumulate in vacuoles of a wide range of plant cells and tissues. They are responsible for orange, brown, red, blue, and purple colors in vegetative and reproductive parts of plants. The scientific interest to the pigments and their uncolored precursors has been attracting by their important functions in plant physiology [1–3] and human health promoting effects [4]. Currently there is a strong tendency to increase anthocyanin content in agricultural plants including cereals [5].

The anthocyanin biosynthesis is universal metabolic pathway that is regulated by transcription factors (TFs) belonging to three families: **my**elo**b**lastosis family of TFs harboring two imperfect amino acid sequence **r**epeats (R2R3-MYB), TFs with basic helix-loop-helix (bHLH) domain that has been described for the first time in the Avian virus oncogene **My**elo**c**ytomatosis (MYC), and TFs carrying structural motif of a tryptophan-aspartic acid dipeptide repeated approximately 40 times (WD40). R2R3-MYB, bHLH (MYC) and WD40 are combined in the MBW complex that regulates expression of the structural genes [6]. With the exception of WD40, which is a universal TF for many cellular processes including flavonoid biosynthesis [7], the other factors are temporally and spatially regulated and activate biosynthesis of anthocyanins or proanthocyanidins in tissue-specific manner [8]. For example, different bHLH and R2R3-MYB factors (in couple with WD40) jointly activate anthocyanin synthesis in vegetative tissues (B1/SN1 and PL1 with PAC1) and seeds (R1 and C1 with PAC1) of maize. In *Arabidopsis* seedlings, anthocyanin biosynthesis is governed by complex constituted by bHLH TT8/GL3/EGL3, R2R3-MYB PAP1/PAP2/MYB113/MYB114, and WD40 TTG1, whereas proanthocyanidins in seeds are synthesized under control of bHLH TT8, R2R3-MYB TT2 and the same WD40 TTG1 (summarized in [8]).

Evolutionary studies have revealed more rapid evolution of the regulatory genes than that of the structural genes encoding biosynthetic enzymes [9]. Variation of pigmentations patters is rather based on the allelic variability of the regulatory genes, than on that of the structural genes. Hence, the identification of the regulatory components of anthocyanin biosynthesis gene network and revealing of features of their own regulation is a paramount task on the way to govern synthesis of the anthocyanin pigments in any plant species.

Barley (*Hordeum vulgare* L.) grain may have yellow, blue, purple color caused by accumulation of flavonoids compounds in distinct layers of grain. Proanthocyanidins synthesized in seed coat gave yellow-colored grains that cannot be distinguished from non-colored white ones. The pigments appear brick red after staining with vanillin-HCl [10]. Accumulation of proanthocyanidins in seed coat is associated with seed dormancy [11]. The proanthocyanidin biosynthesis is governed by a specific R2R3-MYB domain protein, encoded by the *Ant28* gene mapped to the long arm of chromosome 3H [12].

Blue color of barley grain is caused by anthocyanins accumulated in aleurone layer [13]. Recently the candidate genes for components of the MBW complex govern the anthocyanin biosynthesis in aleurone layer have been identified [14]. The *HvMyc2* and *HvMpc2* genes encoding, respectively, bHLH and R2R3-MYB TFs were mapped on the long arm of chromosome 4HL. *HvMyc2* was transcriptionally active in aleurone only, whereas *HvMpc2* was expressed in different tissues, but its transcription was not detected in non-coloured aleurone. Based on homology search through barley genome the *HvWD40* gene was identified as candidate gene for anthocyanin/proanthocyanidin regulatory factor. The gene was located in chromosome 6HL and was expressed constantly in diverse tissues independently on their color [14].

The purple colour of grain pericarp is caused by anthocyanins accumulated in pericarp cells [15]. Recently, two quantitative trait loci (QTLs) controlling individual anthocyanins in purple barley grains were located on chromosomes 2HL and 7HS similar to the position of the *Ant2* and *Ant1* genes, respectively [16], which determine red pigmentation of stem, auricle, awn, and lemma [17]. Increased transcriptional level of the *Ant2* gene in purple pericarp of barley grain [18] and data on its orthologues from other cereals [19, 20] allowed suggesting that *Ant2* controls anthocyanin biosynthesis in pericarp too. The functional role of the *Ant1* gene in barley pericarp pigmentation has not been assessed yet.

To determine role of each of the *Ant* genes in anthocyanin biosynthesis in barley pericarp and reveal possible interactions between the genes we have “split” the purple-grained line carrying the donor’s fragments located in chromosomes 2H (with the dominant allele of the *Ant2* gene) and 7H (with the dominant allele of the *Ant1* gene conferring purple leaf sheath colour) into two lines: one with the donor’s fragment in 2H, and another with the donor’s fragment in 7H. Morphological (red stem, *Rs*, synonymous to *Ant1*) and molecular (*Ant1*- and *Ant2*-specific as well as 7H- and 2H-specific microsatellite) markers were used for selection of plants with desired genotypes.

## Results

### Marker-assisted development of NILs

**Fig. 1:**
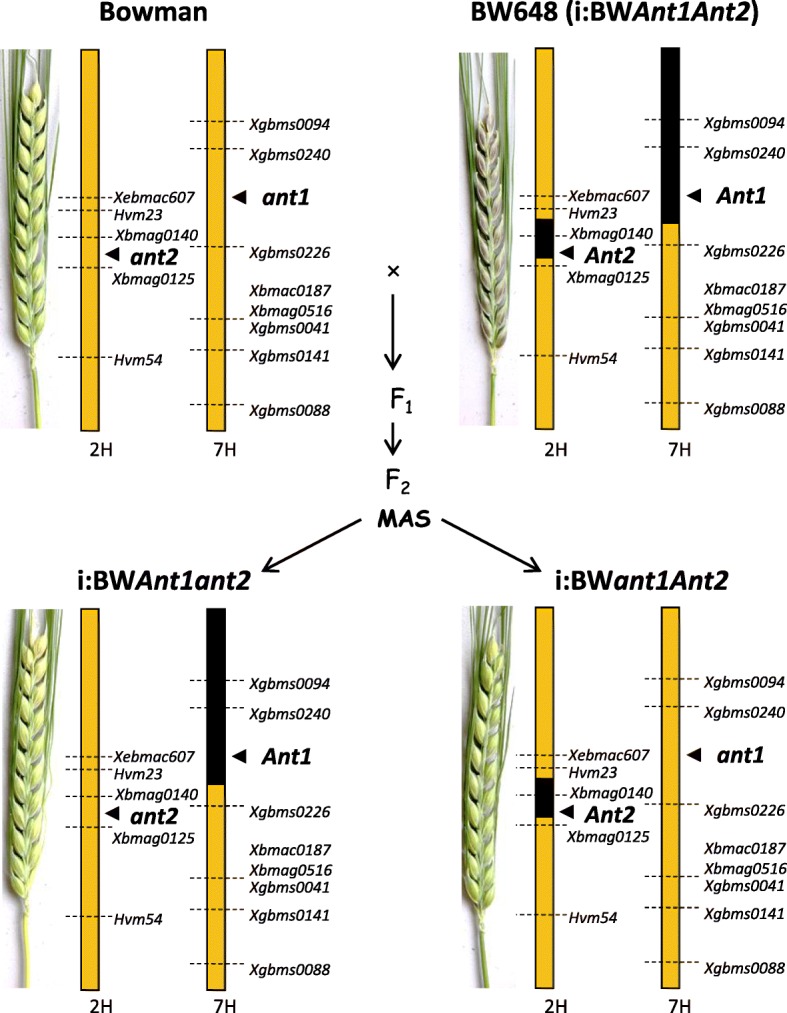
Markers-assisted backcrossing scheme used to obtain and validate NILs carrying different combinations of dominant and recessive alleles of the *Ant* genes. MAS – marker-assisted selection

**Fig. 2:**
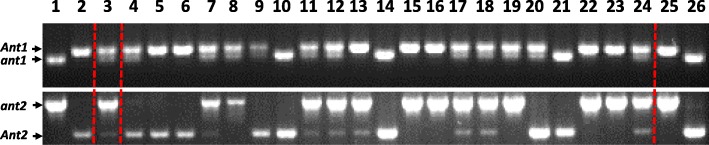
Genotyping of Bowman (line 1), BW648 line (2), their F_1_ (3), F_2_ progenies (4–24) and developed lines i:BW*Ant1ant2* (25) and i:BW*ant1Ant2* (26) by co-dominant markers to the *Ant1* and *Ant2* genes

Bowman (BW) plants with recessive *ant1*, *ant2* were crossed with BW648 having dominant *Ant1* and *Ant2* (Fig. 1); pericarps of the F_1_ hybrid plants were dark purple (Additional file 1). F_2_ progenies with red leaf sheaths (genotype *Ant1Ant1* or *Ant1ant1*) were genotyped using microsatellites flanking the *Ant1* and *Ant2* genes in addition to primers amplifying parts of the *Ant* genes; homozygous plants *Ant1Ant1ant2ant2* were selected. F_2_ progenies with green leaf sheaths (genotype *ant1ant1*) were used to select plants with homozygous genotype *ant1ant1Ant2Ant2* (Fig. 2). Selected F_2_ genotypes that were simultaneously homozygous for BW648 chromosome 7H and BW chromosome 2H microsatellite alleles were designated as i:BW*Ant1ant2*. The genotypes homozygous for BW chromosome 7H and BW648 chromosome 2H microsatellite alleles were designed as i:BW*ant1Ant2*. The parental line BW648 was renamed as i:BW*Ant1Ant2*. Both lines i:BW*Ant1ant2* and i:BW*ant1Ant2* did not accumulate anthocyanins in pericarp (Fig. 1). Line i:BW*Ant1ant2* exhibited pigmented leaf sheaths in contrast to i:BW*ant1Ant2*, that did not accumulate anthocyanins in this part of plant.

### Concentration of total anthocyanins in barley grains

**Fig. 3:**
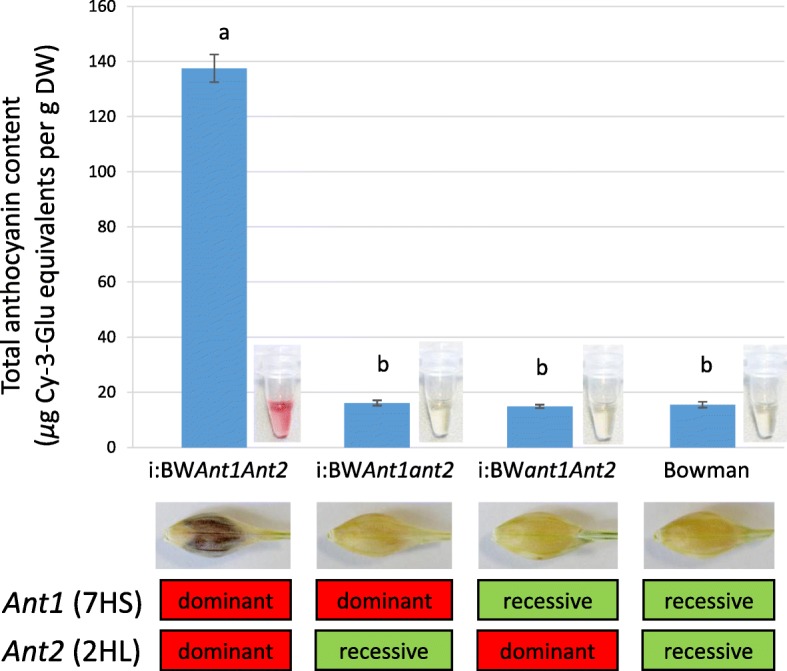
Total anthocyanin content in mature grains of Bowman and its NILs i:BW*Ant1Ant2* (BW648), i:BW*Ant1ant2*, i:BW*ant1Ant2*. The different letters mean statistically significant differences between the lines (*U*-test, *p* ≤ 0.05). DW – dry weight

The total anthocyanin content in grain of the parental (BW, i:BW*Ant1Ant2*) and newly created (i:BW*Ant1ant2*, i:BW*ant1Ant2*) lines was measured (Fig. 3). The i:BW*Ant1Ant2* line carrying dominant alleles of the both *Ant* genes accumulated about 9-fold more anthocyanins in grain than its sister lines having one or both recessive alleles of the *Ant* genes. The detection of anthocyanins in uncoloured lines can be explained by absorbance at 530 nm of other uncoloured compounds.

### Transcription of anthocyanin biosynthesis genes in lemma and pericarp of the NILs

**Fig. 4:**
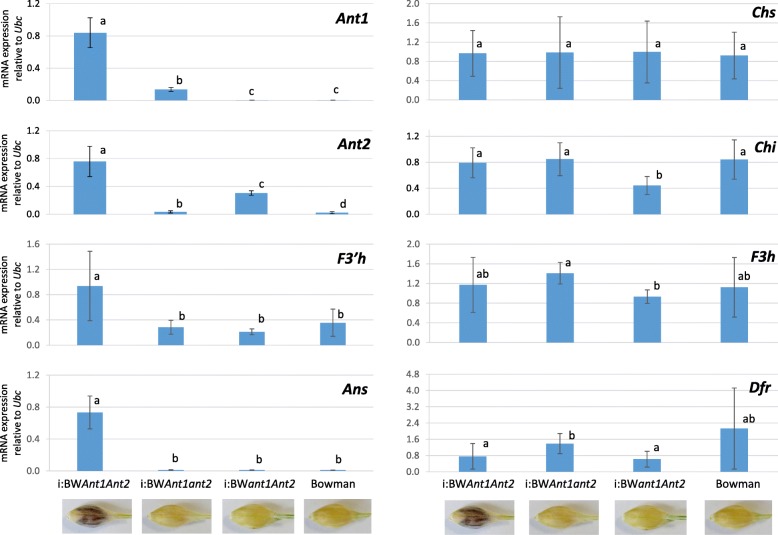
Expression of the anthocyanin biosynthesis genes in lemma and pericarp of the barley NILs with different alleles of the *Ant1* and *Ant2* genes. The data are presented as mean ± standard deviation. The different letters mean statistically significant differences between the lines (*U*-test, *p* ≤ 0.05)

The qRT-PCR-based evaluation of transcript abundance of the regulatory (*Ant1* and *Ant2*) and structural (*Chs*, *Chi*, *F3 h*, *F3 h*, *Dfr*, and *Ans*) genes in lemma and pericarp of the NILs is summarized in Fig. 4.

The transcription of the *Ant* genes was barely detected in lemma and pericarp of the lines harboring recessive alleles of the genes i.e. *Ant1* expression was reduced in Bowman (*ant1ant1ant2ant2*) and i:BW*ant1Ant2*, as well as *Ant2* – in Bowman and i:BW*Ant1ant2*. Relative to Bowman, expression of the *Ant1* and *Ant2* genes was increased, respectively, 71- and 14-fold in lines i:BW*Ant1ant2* and i:BW*ant1Ant2* bearing dominant alleles of the corresponding genes and recessive ones of their counterparts. Significant enhance of the *Ant1* and *Ant2* genes expression in 6- and 2.5 times relative to i:BW*Ant1ant2* and i:BW*ant1Ant2*, respectively, was detected in line i:BW*Ant1Ant2*, where both of the genes are dominant. These data allowed suggesting existence of a reciprocal positive regulatory loop between the *Ant1* and *Ant2* genes.

Analysis of structural genes expression revealed two differently regulated units. The first regulatory unit combines the genes *Chs*, *Chi*, *F3h*, and *Dfr* that transcribed in lines with any combinations of the *Ant* genes (Fig. 4). The differences in expression level of the structural genes were not detected between the lines with exception of decreased expression of *Chi* and *F3 h* in i:BW*ant1Ant2* relative to some other lines and increased expression of the *Dfr* gene in i:BW*Ant1ant2* relative to i:BWa*nt1Ant2* and i:BW*Ant1Ant2* (Fig. 4).

The second regulatory unit represents the *F3’h* and *Ans* genes, that were up-regulated in the line i:BW*Ant1Ant2* only, whereas the other combinations of the *Ant* genes did not affect expression of these genes.

## Discussion

### *Ant1* and *Ant2* are components of the anthocyanin biosynthesis regulatory network in barley grain pericarp

By segregation analysis both monogenic and digenic control has been referred for the purple pigmentation trait [21, 22]. Applying marker-assisted selection dominant alleles of the *Ant1* and *Ant2* genes were split into distinct lines and morphological and chemical analysis of the lines has demonstrated that the both factors are required for anthocyanin synthesis in barley grain (Figs. 1 and 3).

Participation of *Ant2* in anthocyanin biosynthesis in lemma, auricles, and awns was demonstrated by Cockram with co-authors [23]. In addition, it was shown that the gene was up-regulated in purple pericarp of the Bowman NIL BW648/i:BW*Ant1Ant2* ([18], Fig. 4). Jia with co-authors mapped the *Pre2* gene controlling purple pericarp near the marker *HvOs04g47170* [24] that is closely linked to the *Ant2* gene [23]. Although the other copies of bHLH-encoding genes were not detected in the vicinity of *Ant2* [23], the authors assumed that the *Pre2* and *Ant2* genes are not synonymous, because the purple- and white-grained cultivars Yuyaohongdamai and ACCA used for mapping *Pre2* have no polymorphic *Ant2* diagnostic marker. The marker was developed based on a 16-nucleotide deletion identified in all white-grained genotypes [23]. The polymorphism was not also detected between ‘white’ Bowman, ‘purple’ BW648/i:BW*Ant1Ant2* [18] and in 38 out of 39 white-grained barley cultivars and accessions from ICG collection GenAgro (Novosibirsk, Russia, http://ckp.icgen.ru/plants/) (data are not present). The data additionally proved that the deletion arose and locally distributed in Europe [23]. Another mutation associated with the ‘white’ *ant2* allele was identified by Shoeva with co-authors [18]. It is an insertion of 179 nt in recessive *ant2* allele. The polymorphism identified was successfully used to develop the NILs in the current study (Table 1, Fig. 2). Overall, the data confirmed that *Ant2* controls anthocyanin biosynthesis in grain pericarp tissue.

**Table 1:** The diagnostic primers, distinguishing the dominant and recessive alleles of the *Ant1* and *Ant2* genes

Gene	Chromo-some	Primer pair (5′ → 3′)	Annealing temperature, °C	PCR product length (nt) in Bowman/BW648	Accessions
*Ant1*	7HS	F: gtttgccaaaggtctatgtga R: atgaaattcaggaaggtcgt	55	223/248	KP265976–79, barke_contig_1832333, bowman_contig_75050, morex_contig_137164
*Ant2*	2HL	F: gccgtgtgtttccttagtt R: cgagccaacaacaagcgagac	55	447/268	KX035100, barke_contig_2804331, bowman_contig_857662, morex_contig_1573231

The primers were designed based on the sequences retrieved from NCBI and IPK Barley BLAST Server (http://webblast.ipk-gatersleben.de/barley_ibsc/viroblast.php)

The second complementary gene required for purple grain trait development in barely is more elusive. In wheat, based on the segregation analysis cluster of genes *Rc*, *Pc*, *Pls*, *Plb*, *Pan*, *Pp-1* controlling anthocyanin pigmentation of coleoptile, culm, leaf sheath, leaf blade, anthers, and pericarp, respectively, was suggested in homoeologous group 7 chromosomes [19]. Applying positional cloning approach *TaC1* and the same gene called as *TaPpm1* were isolated as candidate genes for *Rc* and *Pp-D1*, respectively [20, 25]. The gene encodes for the R2R3-MYB TF and it was the only R2R3-MYB-encoding gene located in position, where the genes cluster was mapped [20, 25–27]. As the wheat *TaC1/TaPpm1* gene is responsible for coleoptile and pericarp pigmentation, we suggested that the barley orthologue *Ant1* that is responsible for pigmentation of leaf sheath [25, 26, 28], is also required for synthesis of anthocyanins in grain pericarp. We showed that the *Ant1* gene is up-regulated in purple pericarp (Fig. 4). The obtained results and data on orthologues genes from other cereals [19, 20, 25] confirmed that *Ant1* controls anthocyanin biosynthesis in pericarp.

### Positive regulatory loop between *Ant1* and *Ant2*

The qRT-PCR analysis of the *Ant1* and *Ant2* genes in the NILs revealed a reciprocal positive regulatory loop between the genes. The *Ant1* and *Ant2* genes were highly up-regulated when their counterparts are dominant (Fig. 4). This allow suggesting that the complex consisting of ANT1 (R2R3-MYB), ANT2 (bHLH) and WD40 intensify significantly transcription of the *Ant1* and *Ant2* genes.

Another type of regulatory interaction between the regulatory genes was detected in wheat. Using analogous lines having different combinations of the *Pp* genes for purple pericarp trait [29], it was showed that the gene product of *Pp-D1/TaMyb-7D* (R2R3-MYB) slightly suppressed the expression of *TaMyc1/Pp3* (bHLH). Here we additionally tested the expression of the *TaMyb-7D* (synonym of *TaC1*, *TaPpm1*) (Additional file 2). In contrast to barley, there was no effect of *TaMyc1* on the *TaMyb-7D* gene expression.

A complicated regulatory network of negative and positive feedback mechanisms controlling expression of anthocyanin regulatory genes has been described in dicot plant species. In *Arabidopsis*, *TT8* appears to be positively regulated by an MBW complex including WD40 TTG1, MYBs TT2/PAP1 and bHLHs TT8 itself or GL3/EGL3 [8]. Similarly, VvMYC1 is involved in a positive feedback regulation of its own expression in *Vitis vinifera* [30]. In *Petunia hybrid*, ectopic expression of *AN2* (R2R3-MYB) in leaves resulted in ectopic expression of *AN1* (bHLH). Similarly, transcription level of *AN1* is severely reduced in anthers of petunias that lack a functional *AN4* allele (R2R3-MYB), suggesting that *AN1* expression may be regulated by the R2R3-MYB factors constituting MBW complex [31].

The complex regulatory loops in anthocyanin biosynthesis regulation revealed in wheat and barley assumed that the phenomenon is more common than was suggested previously, although some species-specific regulatory features occur.

### Regulation of the anthocyanin biosynthesis structural genes

Analysis of the structural gene expression revealed that the genes of the anthocyanin biosynthesis were differently regulated during anthocyanin biosynthesis in lemma and pericarp of barley. Expression of *Chs*, *Chi*, *F3h*, *Dfr* was not affected by *Ant1* or *Ant2* or their combinations, whereas transcription of *F3’h* and *Ans* was up-regulated significantly when the both *Ant* genes were dominant. This observation is distinct from previously published data that showed co-regulation of the whole set of the genes in purple-grained barley [18, 32]. One of the possible explanations is participation of the structural genes in biosynthesis of other flavonoids like proanthocyanidins that accumulated in barley seed coat [10] and required all enzymes to be active. The peeling procedure used to obtain the lemma and pericarp tissue could not avoid the seed coat material, and the genes, that active in this tissue could be monitored by qRT-PCR. Another possible explanation is that RNA in the studies mentioned was extracted in another stage when the genes had expression patterns distinct from that observed in the current investigation.

## Conclusions

In the current study, applying marker-assisted backcrossing approach we developed a set of near isogenic lines (NILs) that represents a proper genetic model for dissection of the purple pigmentation trait of barley grain. Due to these NILs, we revealed specific features of the anthocyanin biosynthesis regulation in barley pericarp. We showed that the both genes *Ant1* and *Ant2* are components of the regulatory network of the anthocyanin biosynthesis. The positive regulatory interaction at transcriptional level between the *Ant* genes predetermines transcriptional activity each of the regulatory genes as well as late anthocyanin biosynthesis structural genes (*F3’h*, *Ans*). The data represent a strong basis for target manipulation with the quantitative anthocyanin content in the barley grain.

## Materials and methods

### Plant material and phenotyping

Cultivar Bowman (BW) (NGB22812, NordGen, https://www.nordgen.org/en/) without anthocyanin pigmentation in lemma, pericarp, and leaf sheath and its near isogenic line BW648 (NGB22213) [33] having purple pericarp and red leaf sheath were used as parental lines for create derivative lines with different combinations of the *Ant* genes. The parental lines were previously genotyped using microsatellite markers [18, 28].

The scheme of the marker-assisted backcrossing approach used is illustrated in Fig. 1.

In plants of F_2_ population derived from the BW × BW648 crossing, anthocyanin pigmentation of leaf sheath was evaluated for selection of dominant *Ant1*. Pericarp pigmentation of each of the F_2_ progeny was scored at full maturity of grain.

### Marker-assisted selection

DNA was extracted from fresh leaves of plants following Plaschke with co-authors [34]. Microsatellite markers from chromosomes 2H (*Hvm23*, *Hvm54*, *Xbmag0125*, *Xbmag0140*, *Xebmac0607*) and 7H (*Xbmac0187*, *Xbmag0516*, *Xgbms0041*, *Xgbms0088*, *Xgbms0094*, *Xgbms0141*, *Xgbms0226*, *Xgbms0240*) [35, 36] were used in PCRs conducted according to [37]. Dr. Marion Röder (IPK-Gatersleben, Germany) kindly provided the primers for the microsatellite loci. Amplicons were separated through 5% ACTGene agarose gels (ACTGene, Inc., Piscataway, NJ, USA).

*Ant1-* and *Ant2-*specific primers (Table 1) distinguishing dominant and recessive alleles of the genes were used for validation of the alleles of *Ant1* and *Ant2* in newly created NILs. The primer pairs were designed using OLIGO software [38].

### Anthocyanin extraction and measurement

Mature seeds were ground in a laboratory grain mill LZM-1 (Zernotechnika, Moscow, Russia) and 1 g of the sample material was homogenized in 10 ml of 1% HCl/MeOH. The mixture was incubated at 4 °C for 12 h and centrifuged at 12,000 rpm for 25 min at 4 °C. Absorbance was measured on a SmartSpecTMPlus spectrophotometer (Bio-Rad Laboratories, Inc., Hercules, CA, USA) at 530 and 700 nm (to correct for haze [39]). A corrected absorbance value was calculated as (A530 – A700). The total anthocyanin content was determined according to [40] and expressed as micrograms of cyanidin 3-glucoside (Cy-3-Glu) equivalents per gram of dry weight (DW) of sample material.

### RNA extraction, reverse transcription and qRT-PCR

RNA was extracted from the pericarp tissue at early dough stage of grain maturity simultaneously for all genotypes using an RNeasy Plant Mini Kit (QIAGEN, Hilden, Germany), then treated with an RNase-Free DNase Set (QIAGEN, Hilden, Germany). Each genotype was represented by three biological replicates. A 1 μg aliquot of RNA was used to prepare single-stranded cDNA by reverse transcription, based on a RevertAidTM kit (Thermo Fisher Scientific Inc., Waltham, MA, USA) and a (dT)_15_ primer. The subsequent qRT-PCR was conducted by using a SYNTOL SYBR Green I kit (Syntol, Moscow, Russia). The primers used for evaluation of transcript abundance of the anthocyanin biosynthesis structural (*Chs*, *Chi*, *F3h*, *F3’h*, *Dfr*, *Ans*) and regulatory (*Ant1* and *Ant2*) genes are summarized in Additional file 3. A fragment of the *Ubc* (ubiquitin) gene sequence was used for reference purposes [41]. Three technical replicates of each reaction were run. Predetermined amounts of cloned cDNA were used to generate standard curves. The differences in transcript abundance between entries were tested using the Mann–Whitney *U*-test (the threshold was *p* ≤ 0.05).

## Additional files

Additional file 1: Seeds and spikes of cv. Bowman (BW), its near isogenic line BW648 and their F_1_ progeny (BW x BW648). (DOCX 650 kb)

Additional file 2: Expression level of the *TaMyb-7D* and *TaMyc1* genes in wheat near isogenic lines with different alleles of the complementary genes *Pp-D1* and *Pp3*, determining anthocyanin pigmentation of grain pericarp. (DOCX 71 kb)

Additional file 3: Primers used in the current study for qRT-PCR. (DOCX 22 kb)

## Abbreviations

bHLH: basic helix-loop-helix
BW: Bowman
DW: Dry weight
MBW: MYB-bHLHL-WD40
MYB: Myeloblastosis
MYC: Myelocytomatosis
NIL: Near isogenic line
QTL: Quantitative trait locus
TF: Transcription factor

## References

[1] Chalker-Scott L (1999). Environmental significance of anthocyanins in plant stress responses. Photochem Photobiol.

[2] Peer WA, Murphy AS, Groteworld E (2006). Flavonoids as signal molecules. The science of flavonoids. Berlin: Spinger.

[3] Landi M, Tattini M, Gould KS (2015). Multiple functional roles of anthocyanins in plant-environment interactions. Environ Exp Bot.

[4] Smeriglio A, Barreca D, Bellocco E, Trombetta D (2016). Chemistry, pharmacology and health benefits of anthocyanins. Phytother Res.

[5] Zhu F (2018). Anthocyanins in cereals: composition and health effects. Food Res Int.

[6] Hichri I, Barrieu F, Bogs J, Kappel C, Delrot S, Lauvergeat V (2011). Recent advances in the transcriptional regulation of the flavonoid biosynthetic pathway. J Exp Bot.

[7] Zhang B, Schrader A (2017). TRANSPARENT TESTA GLABRA 1-dependent regulation of flavonoid biosynthesis. Plants.

[8] Petroni K, Tonelli C (2011). Recent advances on the regulation of anthocyanin synthesis in reproductive organs. Plant Sci.

[9] Rausher MD, Miller RE, Tiffin P (1999). Patterns of evolutionary rate variation among genes of the anthocyanin biosynthetic pathway. Mol Biol Evol.

[10] Aastrup S, Outtrup H, Erdal K (1984). Location of the proanthocyanidins in the barley grain. Carlsb Res Commun.

[11] Debeaujon I, Léon-Kloosterziel KM, Koornneef M (2000). Influence of the testa on seed dormancy, germination, and longevity in *Arabidopsis*. Plant Physiol.

[12] Himi E, Yamashita Y, Haruyama N, Yanagisawa T, Maekawa M, Taketa S (2011). *Ant28* gene for proanthocyanidin synthesis encoding the R2R3 MYB domain protein (*Hvmyb10*) highly affects grain dormancy in barley. Euphytica.

[13] Faris D.G. Physiol Genet of the kernel color of barley. Doctoral dissertation, University of British Columbia, 1955.

[14] Strygina KV, Börner A, Khlestkina EK (2017). Identification and characterization of regulatory network components for anthocyanin synthesis in barley aleurone. BMC Plant Biol.

[15] Mullick DB, Faris DG, Brink VC, Acheson RM (1958). Anthocyanins and anthocyanidins of the barley pericarp and aleurone tissues. Can J Plant Sci.

[16] Zhang X-W, Jiang Q-T, Wei Y-M, Liu C (2017). Inheritance analysis and mapping of quantitative trait loci (QTL) controlling individual anthocyanin compounds in purple barley (*Hordeum vulgare* L.) grains. PLoS One.

[17] Jende-Strid B, Lundqvist U (1978). Diallelic tests of anthocyanin-deficient mutants. Barley Genet Newsl.

[18] Shoeva OY, Mock H-P, Kukoeva TV, Börner A, Khlestkina EK (2016). Regulation of the flavonoid biosynthesis pathway genes in purple and black grains of *Hordeum vulgare*. PLoS One.

[19] Khlestkina EK (2013). Genes determining coloration of different organs in wheat. Russ J Genet Appl Res.

[20] Jiang W, Liu T, Nan W, Jeewani DC, Niu Y, Li C, Wang Y, Shi X, Wang C, Wang J, Li Y, Gao X, Wang Z (2018). Two transcription factors *TaPpm1* and *TaPpb1* co-regulate anthocyanin biosynthesis in purple pericarps of wheat. J Exp Botany.

[21] Myler JL, Stanford EH (1942). Color inheritance in barley. J Am Soc Agron.

[22] Woodward RW, Thieret JW (1953). A genetic study of complementary genes for purple lemma, Palea and pericarp in barley (*Hordeum vulgare* L.). Agron J.

[23] Cockram J, White J, Zuluaga DL, Smith D, Comadran J, Macaulay M, Luo Z, Kearsey MJ, Werner P, Harrap D, Tapsell C, Liu H, Hedley PE, Stein N, Schulte D, Steuernagel B, Marshall DF, Thomas WT, Ramsay L, Mackay I, Balding DJ (2010). The AGOUEB consortium, Waugh R, O’Sullivan DM. Genome-wide association mapping to candidate polymorphism resolution in the unsequenced barley genome. Proc Natl Acad Sci U S A.

[24] Jia Q, Zhu J, Wang J, Yang J, Zhang G (2016). Genetic mapping and molecular marker development for the gene *Pre2* controlling purple grains in barley. Euphytica.

[25] Himi E, Taketa S (2015). Isolation of candidate genes for the barley *Ant1* and wheat *Rc* genes controlling anthocyanin pigmentation in different vegetative tissues. Mol Gen Genomics.

[26] Zakhrabekova S, Dockter C, Ahmann K, Braumann I, Gough SP, Wendt T, Lundqvist U, Mascher M, Stein N, Hansson M (2015). Genetic linkage facilitates cloning of *Ert-m* regulating plant architecture in barley and identified a strong candidate of *Ant1* involved in anthocyanin biosynthesis. Plant Mol Biol.

[27] Strygina KV, Khlestkina EK. Structural and functional divergence of the *TaMpc1* genes in wheat and barley. BMC Evol Biol. 2019;in press. 10.1186/s12862-019-1378-3 PMC6391766 30813913

[28] Shoeva OY, Kukoeva TV, Börner A, Khlestkina EK (2015). Barley *Ant1* is a homolog of maize *C1* and its product is part of the regulatory machinery governing anthocyanin synthesis in the leaf sheath. Plant Breed.

[29] Gordeeva EI, Shoeva OY, Khlestkina EK (2015). Marker-assisted development of bread wheat near-isogenic lines carrying various combinations of purple pericarp (*Pp*) alleles. Euphytica.

[30] Hichri I, Heppel SC, Pillet J, Leon C, Czemmel S, Delrot S, Lauvergeat V, Bogs J (2010). The basic helix–loop–helix transcription factor MYC1 is involved in the regulation of the flavonoid biosynthesis pathway in grapevine. Mol Plant.

[31] Albert NW, Davies KM, Lewis DH, Zhang H, Montefiori M, Brendolise C, Boase MR, Ngo H, Jameson PE, Schwinn KE (2014). A conserved network of transcriptional activators and repressors regulates anthocyanin pigmentation in eudicots. Plant Cell.

[32] Meldgaard M (1992). Expression of chalcone synthase, dihydroflavonol reductase, and flavanone-3-hydroxylase in mutants of barley deficient in anthocyanin and proanthocyanidin biosynthesis. Theor Appl Genet.

[33] Druka A, Franckowiak J, Lundqvist U, Bonar N, Alexander J, Houston K, Radovic S, Shahinnia F, Vendramin V, Morgante M, Stein N, Waugh R (2011). Genetic dissection of barley morphology and development. Plant Physiol.

[34] Plaschke J, Ganal MW, Röder MS (1995). Detection of genetic diversity in closely related bread wheat using microsatellite markers. Theor Appl Genet.

[35] Li JZ, Sjakste TG, Röder MS, Ganal MW (2003). Development and genetic mapping of 127 new microsatellite markers in barley. Theor Appl Genet.

[36] Ramsay L, Macaulay M, degli Ivanissivich S, Maclean K, Cardle L, Fuller J, Edwards K, Tuvensson S, Morgante M, Massari A, Maesti E, Marmiroli N, Sjakste T, Ganal M, Powell W. Waugh R A simple sequence repeat-based linkage map of barley. Genetics, 2000;156:1997–2005. 10.1093/genetics/156.4.1997 PMC1461369 11102390

[37] Röder MS, Korzun V, Wendehake K, Plaschke J, Tixier M-H, Leroy P, Ganal MW (1998). Microsatellite map of wheat. Genetics.

[38] Offerman JD, Rychlik W, Krawetz SA, Womble DD (2003). Oligo primer analysis software. Introduction to bioinformatics: a theoretical and practical approach.

[39] Giusti MM, Wrolstad RE, Wrolstad RE, Acree TE, An H, Decker EA, Penner MH, Reid DS, Schwartz SJ, Shoemaker CF, Sporns P (2001). Characterization and measurement of anthocyanins by UV-visible spectroscopy. Current protocols in food analytical chemistry.

[40] Abdel-Aal E-SM, Hucl P (1999). A rapid method for quantifying total anthocyanins in blue aleurone and purple pericarp wheats. Cereal Сhem.

[41] Himi E, Nisar A, Noda K (2005). Colour genes (*R* and *Rc*) for grain and coleoptile upregulate flavonoid biosynthesis genes in wheat. Genome.

