# Supplementary material for: Purple-grained barley (Hordeum vulgare L.): marker-assisted development of NILs for investigating peculiarities of the anthocyanin biosynthesis regulatory network
Source: BMC Plant Biol. 2019 Feb 15;19(Suppl 1):52. doi: 10.1186/s12870-019-1638-9 (PMC6393963; doi:10.1186/s12870-019-1638-9)

**Additional file 1.** Seeds and spikes of parental cv. Bowman (BW), near isogenic line BW648, and their F_1_ progeny (BW x BW648).


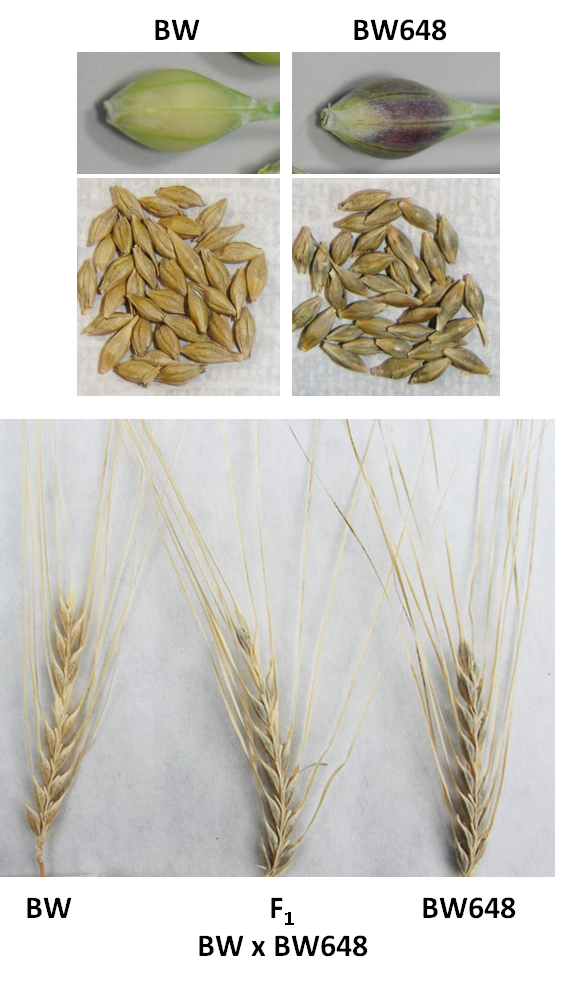

Supplement: Supplementary file 1 — Seeds and spikes of cv. Bowman (BW), its near isogenic line BW648 and their F1 progeny (BW x BW648). (DOCX 650 kb) [file 12870_2019_1638_MOESM1_ESM.docx]
