# Supplementary material for: Purple-grained barley (Hordeum vulgare L.): marker-assisted development of NILs for investigating peculiarities of the anthocyanin biosynthesis regulatory network
Source: BMC Plant Biol. 2019 Feb 15;19(Suppl 1):52. doi: 10.1186/s12870-019-1638-9 (PMC6393963; doi:10.1186/s12870-019-1638-9)
Supplement: Supplementary file 2 — Expression level of the TaMyb-7D and TaMyc1 genes in wheat near isogenic lines with different alleles of the complementary genes Pp-D1 and Pp3, determining anthocyanin pigmentation of grain pericarp. (DOCX 71 kb) [file 12870_2019_1638_MOESM2_ESM.docx]

| 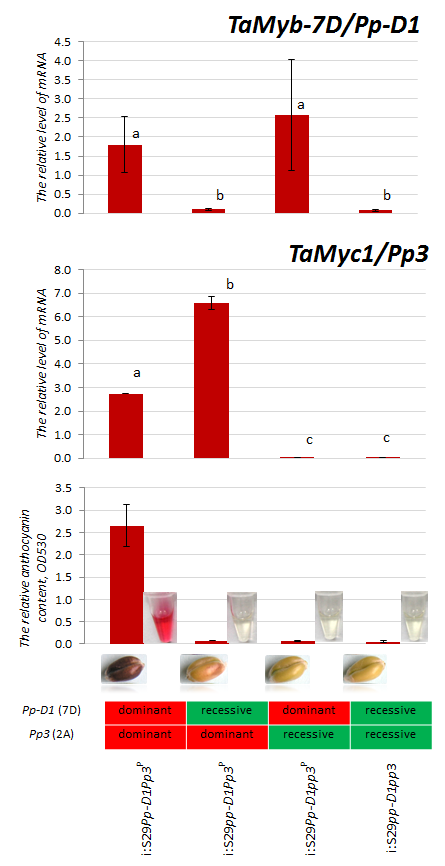 | **Additional file 2.** Expression level of the *TaMyb-7D* and *TaMyc1* genes in wheat near isogenic lines with different alleles of the complementary genes *Pp-D1* and *Pp3*, determining anthocyanin pigmentation of grain pericarp. The lines were developed by Gordeeva et al. (2015). Expression of *TaMyc1* in the created lines was monitored earlier (Shoeva et al. 2014). Expression of the *TaMyb-7D* was assessed in the current study using cDNA from previous investigation (Shoeva et al. 2014) and primers (Forward 5’gaggagatcaagagggagac3’, Reverse 5’catgtgcagggacttgag3’) amplifying part of the *TaC1-D1* (AB983542) gene identified as gene candidate for *Rc* (red coleoptile) (Himi and Taketa 2015). The *TaC1-D1* gene is identical to *TaPpm1*, encoding R2R3-MYB transcriptional factor regulating with its bHLH counterpart from chromosome 2A anthocyanin biosynthesis in wheat pericarp (Jiang et al. 2018).  **References**  Gordeeva EI, Shoeva OY, Khlestkina EK. Marker-assisted development of bread wheat near-isogenic lines carrying various combinations of purple pericarp (*Pp*) alleles. Euphytica. 2015;203(2):469-476. doi:10.1007/s10681-014-1317-8.  Himi E, Taketa S. Isolation of candidate genes for the barley *Ant1* and wheat *Rc* genes controlling anthocyanin pigmentation in different vegetative tissues. Mol. Genet. Genomics. 2015;290(4):1287-1298. doi: 10.1007/s00438-015-0991-0.  Jiang W, Liu T, Nan W, Jeewani DC, Niu Y, Li C, Wang Y, Shi X, Wang C, Wang J, Li Y, Gao X, Wang Z. Two transcription factors *TaPpm1* and *TaPpb1* co-regulate anthocyanin biosynthesis in purple pericarps of wheat. J Exp Botany. 2018;69(10):2555–2567. doi:10.1093/jxb/ery101.  Shoeva OY, Gordeeva EI, Khlestkina EK. The regulation of anthocyanin synthesis in the wheat pericarp. Molecules. 2014;19:20266–20279. doi: 10.3390/molecules191220266. |
| --- | --- |
