# Supplementary material for: Purple-grained barley (Hordeum vulgare L.): marker-assisted development of NILs for investigating peculiarities of the anthocyanin biosynthesis regulatory network
Source: BMC Plant Biol. 2019 Feb 15;19(Suppl 1):52. doi: 10.1186/s12870-019-1638-9 (PMC6393963; doi:10.1186/s12870-019-1638-9)
Supplement: Supplementary file 3 — Primers used in the current study for qRT-PCR. (DOCX 22 kb) [file 12870_2019_1638_MOESM3_ESM.docx]

**Additional file 3.** Primers used in the current study for qRT-PCR analysis

| Gene | NCBI accessions or IPK contigs or references | Forward primer (5’→3’) | Reverse primer (5’→3’) | PCR-product length (DNA/cDNA), nt | Annealing temperature, °C | Reference |
| --- | --- | --- | --- | --- | --- | --- |
| *Chs* | X58339 | aagatcaccaagagcgacca | cgacgacgacgatgtcct | 1467/178 | 55 | [1] |
| *Chi* | AF474923 | cggacaaggtgacggagaa | ggagaaggcgacggtgag | 176/176 | 55 | [1] |
| *F3h* | X58138 | caccataacgctcctcct | gcctgctgctctccc | 303/194 | 55 | [1] |
| *F3’h* | AK363912 | gccagggagttcaaggaca | ctcgctgatgaatccgtcca | 168/168 | 55 | [2] |
| *Dfr* | S69616 | gcgtcgggtttcgtagggtc | cgcgatggcctcgttgaag | 309/192 | 60 | [1] |
| *Ans* | 941389* | gggcctgcaggtcgtcaaca | ggcgcagcagcacggagtc | 209/209 | 60 | current study |
| *Ant1* | KP265976-79 | ggagaagagccaccatttc | gtcgtccatccagtctcc | 108/108 | 55 | current study |
| *Ant2* | KX035100 | gagatcaacgggttctacgg | gagtcccacgatctcttcca | 215/215 | 60 | [3] |
| *Ubiquitin* | AY297059 | atttgtgaagaccctcaccg | caccaagtgaagggtggact | 201/201 | 60 | [4] |

* contig of cv. Bowman found at IPK Barley BLAST Server (http://webblast.ipk-gatersleben.de/barley_ibsc/viroblast.php)

**References**

1. Shoeva OY, Kukoeva TV, Börner A, Khlestkina EK. Barley *Ant1* is a homolog of maize *C1* and its product is part of the regulatory machinery governing anthocyanin synthesis in the leaf sheath. Plant Breed. 2015;134(4):400-405. doi:10.1111/pbr.12277.

2. Shoeva OY., Mock H-P, Kukoeva TV, Börner A, Khlestkina EK Regulation of the flavonoid biosynthesis pathway genes in purple and black grains of *Hordeum vulgare*. PLoS ONE. 2016;11(10):e0163782. doi:10.1371/journal. pone.0163782.

3. Cockram J, White J, Zuluaga DL, Smith D, Comadran J, Macaulay M, et al. Genome-wide association mapping to candidate polymorphism resolution in the unsequenced barley genome. Proc Natl Acad Sci U S A. 2010;107(50):21611-21616. doi: 10.1073/pnas.1010179107 PMID: 21115826.

4. Himi E, Nisar A, Noda K. Colour genes (*R* and *Rc*) for grain and coleoptile upregulate flavonoid biosynthesis genes in wheat. Genome. 2005;48:747-754. doi:10.1139/g05-026 PMID: 16094442.
